# Navigating neural pathways: how stimulation polarity shapes deep brain stimulation efficacy

**DOI:** 10.1093/braincomms/fcaf061

**Published:** 2025-02-08

**Authors:** Atefeh Asadi, Nabin Koirala, Muthuraman Muthuraman

**Affiliations:** Department of Neurology, University Hospital Würzburg, Würzburg 97080, Germany; Brain Imaging Research Core, University of Connecticut, Storrs, CT 06269, USA; School of Medicine, Yale University, New Haven, CT 06511, USA; Center for Biomedical Imaging & Neuromodulation, Nathan Kline Institute for Psychiatric Research, Orangeburg, NY 10962, USA; Department of Neurology, University Hospital Würzburg, Würzburg 97080, Germany; Informatics for Medical Technology, Institute of Computer Science, University Augsburg, Augsburg 86159, Germany

## Abstract

This scientific commentary refers to ‘Neural pathway activation in the subthalamic region depends on stimulation polarity’ by Borgheai *et al*. (https://doi.org/10.1093/braincomms/fcaf006).

**This scientific commentary refers to ‘Neural pathway activation in the subthalamic region depends on stimulation polarity’ by Borgheai *et al*. (https://doi.org/10.1093/braincomms/fcaf006).**

Deep brain stimulation (DBS) is an evidence-based therapy for symptomatic treatment of patients with Parkinson's disease and is shown to be primarily effective for managing motor symptoms. During DBS, a device is implanted in a specific brain region [subthalamic nucleus (STN) and globus pallidum (GP) are the most used targets for Parkinson’s disease], which send electrical signals in a specific polarity and waveform to modulate the symptoms. Although the exact underlying mechanism of DBS is still under debate, it has been shown that parameters used during the stimulation plays a vital role in alleviating specific symptoms.^1^

Parkinson’s disease is associated with widespread neurodegeneration within the basal ganglia—a group of subcortical nuclei that, together with cortex and thalamus form the cortico- basal ganglia -thalamo-cortical loop. According to the classical model of basal ganglia,^2^ motor control relies on the balance between two striatal pathways—direct and indirect and medium-sized spiny neurons in the striatum are divided into two distinct populations defining these pathways (see Fig. 1). One group of medium-sized spiny neurons, expressing D1 dopamine receptors^3,4^ utilizes dopamine delivered by substantia nigra pars compacta as an excitatory neurotransmitter and projects directly to the GPi/SNr (globus pallidus internus/substantia nigra pars reticulata) and make the direct pathway. This pathway is thought to facilitate movement initiation and execution.^5^ Whereas, the medium-sized spiny neurons which express D2 dopamine receptor^3,4^ and use the dopamine from substantia nigra pars compacta as an inhibitory neurotransmitter projects to globus pallidus externus (GPe) and relay their outputs to GPi/SNr via GPe and subthalamic nucleus (STN; Fig. 1). This indirect pathway in patients with Parkinson’s disease is believed to become hyperactive and inhibit movement.^5^ In addition to the direct and indirect pathways, the basal ganglia circuitry includes the hyperdirect pathway, which plays a critical role in motor control by rapidly inhibiting motor responses. Unlike the direct and indirect pathways, where the striatum serves as the primary input structure, the hyperdirect pathway bypasses the striatum, with the STN acting as the input nucleus (Fig. 1). Through fast excitatory input from the cortex to the STN, it subsequently increases inhibitory output from the GPi/SNr to the thalamus, effectively suppressing movement.^6^

**Figure 1:**
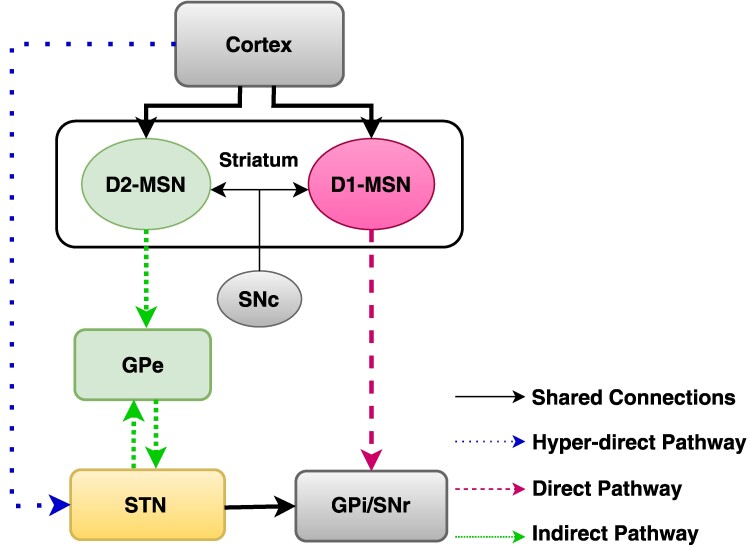
**Schematic of the pathways in the cortico-basal ganglia network.** See text for explanations.

The basal ganglia can also be conceptualized through the centre-surround model, a conceptual framework, which describes how the mediate action selection by facilitating intended actions while simultaneously suppressing competing or unintended movements. In Parkinson’s disease, the degeneration of dopaminergic neurons disrupts the balance of these basal ganglia pathways, often resulting in excessive activation of the hyperdirect pathway, which may exacerbate Parkinson’s disease symptoms by further increasing activity in the STN and GPi, leading to heightened movement suppression.^7^ However, the centre-surrounded model suggests that the hyperdirect pathway may also play a compensatory role by suppressing competing actions^8^ with some evidence to suggests that optical stimulation of cortico-STN terminals may hold therapeutic potential in alleviating Parkinson’s disease symptoms.^9^ However, it should be noted that the outcome of activating the hyperdirect pathway is complex and can vary depending on the specific parameters of the activation and the state of the basal ganglia circuitry.^7^

In this issue of *Brain Communications*, Borgheai *et al*.^10^ study the effects of polarity of the stimulus waveform (cathodic versus anodic) on the activation of neural pathways in the subthalamic region during DBS in patients with Parkinson’s disease. The primary aim of the study was to assess how stimulation parameters impact the recruitment of clinically relevant pathways, which might help us understand the biophysical mechanism of DBS and in general, electric stimulation in the brain. By recording cortical evoked potentials using subdural electrocorticography, DBS local evoked potentials from non-stimulating contacts, and electromyography activity from arm and face muscles, the researchers compared the activation of the cortico-STN hyperdirect pathway, the STN-GP pathway, and the cortico-spinal/bulbar tract during cathodic and anodic DBS.

The key finding of Borgheai *et al*.^10^ is that both anodic and cathodic stimulation activates the same pathways, but cathodic stimulation was generally more efficient in activating STN pathways, evoking significantly larger response amplitudes for most evoked potentials. However, DBS local evoked potentials amplitudes did not significantly differ between the two polarities and when comparing the relative efficiency, anodic stimulation was least efficient in activating the cortico-spinal/bulbar tract, moderately efficient for the hyperdirect pathway, and equally efficient to cathodic stimulation for the STN-GP pathway. While the latencies of cathodic and anodic responses were in the same range, anodic stimulation exhibited slight delays (100–500 μs) for some evoked potentials. Additionally, anodic stimulation required approximately twice the current as cathodic stimulation to achieve comparable activation of the hyperdirect pathway. These findings aligned with computational models, confirming pathway-specific differences in excitability metrics and similar chronaxie values for both polarities, indicative of activation of myelinated fibres.

Clinically, these findings have important implications for optimizing DBS therapy by examining the differential efficiency of anodic and cathodic stimulation in activating specific pathways. This difference help explain why anodic DBS may widen the therapeutic window in clinical practice, as it is less efficient in activating the cortico-spinal/bulbar tract, potentially reducing side effects. The study provides evidence supporting the role of hyperdirect pathway in therapeutic benefits, as its activation required more anodic current, while the STN-GP pathway responded similarly to both polarities. Moreover, these findings are consistent with prior studies suggesting that antidromic activation of hyperdirect pathway alters cortical activity and can alleviate parkinsonian motor deficits.^8^ These results emphasize the potential to refine DBS strategies by selectively targeting pathways to maximize therapeutic benefits while minimizing side effects. However, the use of low-frequency stimulation in this study, as opposed to standard clinical high-frequency DBS, may limit generalizability. Evidence suggesting that low-frequency stimulation of the STN during DBS, which could potentially enhance beta oscillatory activity in the STN or its connected networks, may worsen bradykinesia.^9,11^ Similar studies in rodents have shown specifically stimulating neurons in the hyperdirect pathway at low frequencies can also exacerbate bradykinesia.^9^ Additionally, previous studies have demonstrated that high- and low-frequency modulation may have different mechanism of actions. Considering these factors together, we urge the readers to interpret the findings with caution when evaluating the role of hyperdirect pathway stimulation in therapeutic benefits.

Taking all results together, the study suggests that DBS modulates the balance between therapeutic and maladaptive pathways within the basal ganglia in Parkinson’s disease. The hyperdirect pathway which serves as a direct link from the cortex to the STN, plays a dual role in Parkinson’s disease—facilitating motor action selection while becoming hyperactive due to dopamine loss, leading to increased STN activity and bradykinesia. The findings also indicate that cathodic DBS is more efficient in activating the hyperdirect pathway, as indicated by larger cortical evoked potentials and lower activation thresholds making it particularly suited for engaging therapeutic pathways. In contrast, anodic stimulation requires higher currents to activate the hyperdirect pathway but offers the advantage of broadening the therapeutic window by reducing activation efficiency in pathways such as the cortico-spinal/bulbar tract, which is associated with motor-related side effects. Furthermore, the STN-GP pathway, known for its involvement in resonant oscillatory activity, demonstrated similar excitability under both cathodic and anodic stimulation, suggesting a potential role in modulating motor symptoms independent of stimulus polarity. These results provide new insights into DBS mechanisms and emphasize the importance of electrode positioning, axonal excitability, and pathway-specific responses to different stimulation polarities. The findings omit cortical motor and sensory contributions but underscores the selective modulation of neural circuits during DBS underscoring the need for future research integrating high-frequency DBS with functional imaging and electrophysiology to validate these hypotheses and refine stimulation strategies. Overall, this study highlights the importance of tailoring DBS parameters to selectively target therapeutic pathways while minimizing off-target effects, offering insights for the development of more precise and effective interventions for Parkinson’s disease motor symptoms.

## Data Availability

The data underlying this article are available in the article and in its online supplementary material.

## References

[1] Muthuraman M , Palotai M, Jávor-Duray B, et al Frequency-specific network activity predicts bradykinesia severity in Parkinson’s disease. Neuroimage Clin. 2021;32:102857. 34662779 10.1016/j.nicl.2021.102857 PMC8526781

[2] Albin RL , Young AB, Penney JB. The functional anatomy of basal ganglia disorders. Trends Neurosci. 1989;12(10):366–375. 2479133 10.1016/0166-2236(89)90074-x

[3] Gerfen CR , Engber TM, Mahan LC, et al D1 and D2 dopamine receptor-regulated gene expression of striatonigral and striatopallidal neurons. Science. 1990;250(4986):1429–1432. 2147780 10.1126/science.2147780

[4] Hervé D , Rogard M, Lévi-Strauss M. Molecular analysis of the multiple golf α subunit mRNAs in the rat brain. Brain Res Mol Brain Res. 1995;32(1):125–134. 7494450 10.1016/0169-328x(95)00070-9

[5] Calabresi P , Picconi B, Tozzi A, Ghiglieri V, Di Filippo M. Direct and indirect pathways of basal ganglia: A critical reappraisal. Nat Neurosci. 2014;17(8):1022–1030. 25065439 10.1038/nn.3743

[6] Nambu A , Tokuno H, Takada M. Functional significance of the cortico–subthalamo–pallidal ‘hyperdirect’ pathway. Neurosci Res. 2002;43(2):111–117. 12067746 10.1016/s0168-0102(02)00027-5

[7] Monakow KH , Akert K, Künzle H. Projections of the precentral motor cortex and other cortical areas of the frontal lobe to the subthalamic nucleus in the monkey. Exp Brain Res. 1978;33:395–403. 83239 10.1007/BF00235561

[8] McGregor MM , Nelson AB. Circuit mechanisms of Parkinson’s disease. Neuron. 2019;101(6):1042–1056. 30897356 10.1016/j.neuron.2019.03.004

[9] Gradinaru V , Mogri M, Thompson KR, Henderson JM, Deisseroth K. Optical deconstruction of parkinsonian neural circuitry. Science. 2009;324(5925):354–359. 19299587 10.1126/science.1167093 PMC6744370

[10] Borgheai SB , Opri E, Isbaine F, et al Neural pathway activation in the subthalamic region depends on stimulation polarity. Brain Commun. 2025:fcaf006. 10.1093/braincomms/fcaf006 PMC11839843 39980742

[11] Timmermann L , Wojtecki L, Gross J, et al Ten-hertz stimulation of subthalamic nucleus deteriorates motor symptoms in Parkinson's disease. Mov Disord. 2004;19(11):1328–1333. 15389990 10.1002/mds.20198

